# Crystallographic plane-orientation dependent atomic force microscopy-based local oxidation of silicon carbide

**DOI:** 10.1186/1556-276X-6-235

**Published:** 2011-03-18

**Authors:** Jung-Joon Ahn, Yeong-Deuk Jo, Sang-Cheol Kim, Ji-Hoon Lee, Sang-Mo Koo

**Affiliations:** 1 School of Electronics and Information, Kwangwoon University, Seoul 139-701, Korea; 2 Korea Electrotechnology Research Institute, Power Semiconductor Research Group, Changwon 641-120, Korea

## Abstract

The effect of crystalline plane orientations of Silicon carbide (SiC) (a-, m-, and c-planes) on the local oxidation on 4H-SiC using atomic force microscopy (AFM) was investigated. It has been found that the AFM-based local oxidation (AFM-LO) rate on SiC is closely correlated to the atomic planar density values of different crystalline planes (a-plane, 7.45 cm^-2^; c-plane, 12.17 cm^-2^; and m-plane, 6.44 cm^-2^). Specifically, at room temperature and under about 40% humidity with a scan speed of 0.5 μm/s, the height of oxides on a- and m-planes 4H-SiC is 6.5 and 13 nm, respectively, whereas the height of oxides on the c-plane increased up to 30 nm. In addition, the results of AFM-LO with thermally grown oxides on the different plane orientations in SiC are compared.

## Introduction

Silicon carbide (SiC) is a well-known wide band gap semiconductor material, which exhibits high values of thermal conductivities, critical fields, and chemical inertness. However, there have been challenges in processing SiC into device applications, since the electric characteristics and yield ratio of SiC-based devices are hampered by micro-pipes and stacking faults. Typical SiC wafers have dislocation densities in the range of 10^3^-10^5 ^cm^-2, ^and in order to prevent this problem, extensive studies on bulk growths, thermal oxidations, and etching properties have been conducted on various crystalline planes in SiC [1-4].

**Figure 1:**
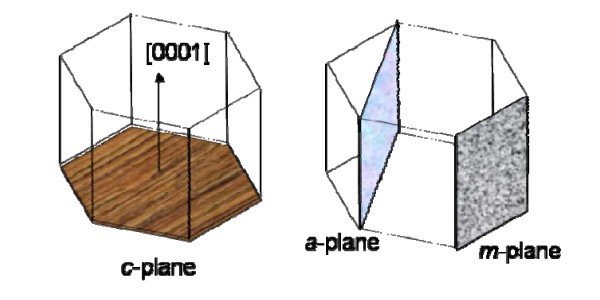
**The crystal structures of c-, a-, and m-planes on 4H-SiC substrates from left to right**.

In recent years, atomic force microscopy-based local oxidation lithography (AFM-LO) techniques have been receiving increasing attention as attractive, emerging lithography techniques for fabrication of nano-scale patterns and related device structures [5-7]. Although electron beam and nano-imprint lithography techniques have been widely studied, there are issues with regard to the damage to structures caused by high-energy electron beams or high imprinting temperatures [8]. On the other hand, AFM-LO can be used as a standard method for the fabrication as well as the characterization of nanostructures and electronic devices, particularly in silicon, since silicon oxides are indispensably used as gate dielectrics, insulation/passivation, and masks. So far, there have been many studies reporting on AFM-LO in various materials [5,9-11]. However, there have been few published studies on AFM-LO of different crystalline planes (a-, c-, and m-planes) of SiC. The enhanced AFM-LO of 4H-SiC at room temperature without heating, chemicals, or photo-illumination has been observed [12]. In this study, the effect of crystalline plane orientations of SiC (a-, m-, and c-planes) on the AFM-LO of SiC was investigated. We compared the rates of AFM-LO and thermal oxidation of horizontal crystalline plane orientations (a- and m-planes) with those of perpendicular crystalline plane orientation (c-plane) to the *c*-axis in 4H-SiC. Figure 1 shows the crystal structures of the c-, a-, and m-planes on 4H-SiC substrates from left to right, respectively [13].

## Experiment

Three different sets of 4H-SiC samples were prepared with corresponding different plane orientations of a- (*N*_D_: 5.9 × 10^18 ^cm^-2^), c- (*N*_D_: 9.6 × 10^18 ^cm^-2^), and m- (*N*_D_: 9.3 × 10^18 ^cm^-2^) planes. AFM (Bruker AXS Inc.)-based local oxidation was performed using the contact mode, whereas the topographic AFM measurement was performed in the non-contact mode AFM. Si cantilevers with a spring constant of 48 N/m, a resonance frequency of 190 kHz, and a radius of 5 nm were used to analyze the morphology of surfaces. For the AFM-LO, Pt/Ir-coated Si conductive tips with radii of 50 nm were used. The spring constant and the resonance frequency were set at 3 N/m and 70 kHz, respectively. The temperature and the humidity of the atmosphere were controlled at 27°C (± 2°C) and 40% (± 5%), respectively, during the AFM-LO process. A dc bias was applied between the cantilever and the substrate for the local oxidation. The electrical field was then created between the native oxide layer and the substrate, which caused the oxyanions (OH^-^) to drift through the oxide film [14-16]. In the case of SiC, the reactions in the AFM-LO were described by the following chemical reactions. In the anode (sample surface), the oxidation takes place as follows: SiC + 2H_2_O + 4h^+ ^→ SiO_2 _+ 4H^+ ^+ C^4+^, SiC + 3/2O_2 _+ 4h^+ ^→ SiO_2 _+ CO↑. The oxyanions (OH^-^) contribute to the formation of the oxide patterns in the surface, while hydrogen generation occurs at the tip (cathode) to complete the electrochemical reaction, 2H^+^(aq) + 2e^- ^→ H_2_. The local oxide patterns were formed on *n*-type a-, m-, and c-planes of c-face 4H-SiC with a doping concentration of 10^19 ^cm^-3^.

## Results and discussion

In general, it is difficult to form oxide patterns on SiC using AFM-LO because of both physical hardness and chemical inactivity. The binding energy of a Si-C bond (451.5 kJ/mol) is higher than that of a Si-Si bond (325 kJ/mol), and thus the reactions of oxyanions (OH^-^) into a Si-C bond require a higher activation energy. The removal of carbon atoms in the forms of CO or CO_2 _species also requires additional energy. The simulation examination that contains the 2D electric field distribution between the tip and both Si and SiC substrates to optimize the doping concentration of materials and the direction of applied bias in oxide formation was carried out. We used ATLAS simulator by Silvaco Inc. to design the tip and semiconductor (SiC or Si) structure with a 10-nm-thick oxide layer and the doping concentration for the semiconductor was varied in the range between 10^15 ^and 10^19 ^cm^-2^.

**Figure 2:**
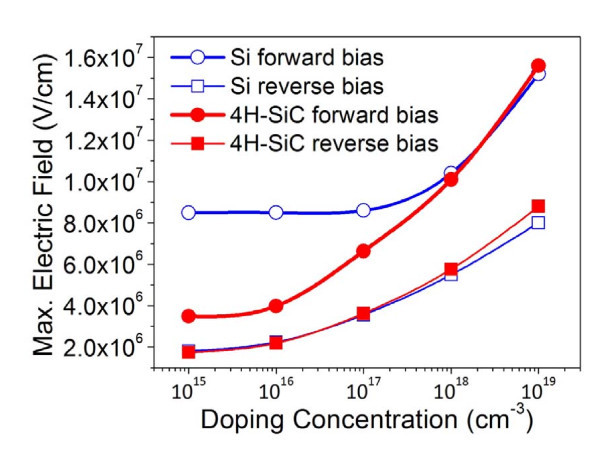
**Simulated maximum electric field values for different doping concentrations (10^15^-10^19 ^cm^-2^) of *n*-type 4H-SiC and Si**.

**Figure 3:**
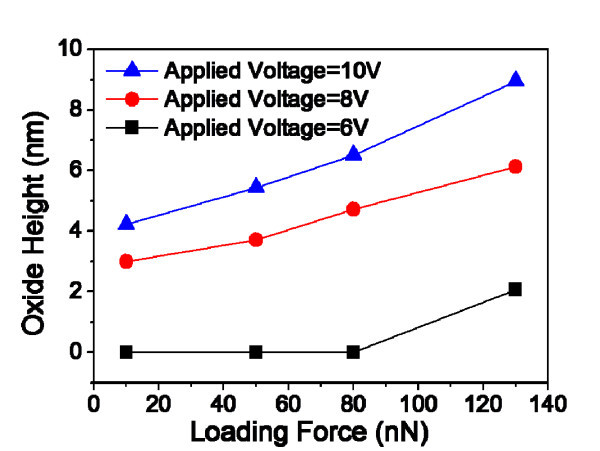
**Variations in AFM-LO oxide height with different loading forces and applied voltages**.

The maximum electric field is located on the tip-contacted surface, and the electric field increases when the doping concentration of the substrates increases, as shown in Figure 2. The electric field enhances the transport of oxyanions (OH^-^, O^2-^) [2], and also the bias direction affects the OH^- ^diffusion across the oxide layer [8]. The variation in oxide height can also be affected by the magnitude of loading force and applied voltage values. Figure 3 represents that the oxide patterns are formed over the LF of 100 nN at an applied voltage of 6 V. The local oxidation rates increase with increasing applied voltages because of the wider effective contact area and higher electric field.

**Figure 4:**
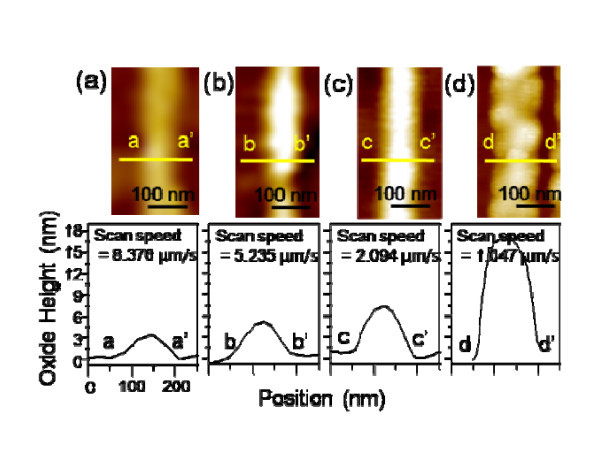
**AFM images and cross-sectional curves of oxide lines on c-plane 4H-SiC obtained under different scan speeds: (a) 8.376 μm/s; (b) 5.235 μm/s; (c) 2.094 μm/s; and (d) 1.047 μm/s**.

**Figure 5:**
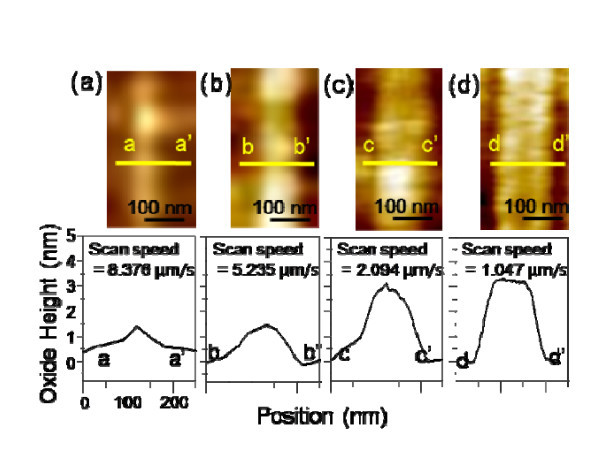
**AFM images and cross-sectional curves of oxide lines on a-plane 4H-SiC obtained under different scan speeds: (a) 8.376 μm/s; (b) 5.235 μm/s; (c) 2.094 μm/s; and (d) 1.047 μm/s**.

**Figure 6:**
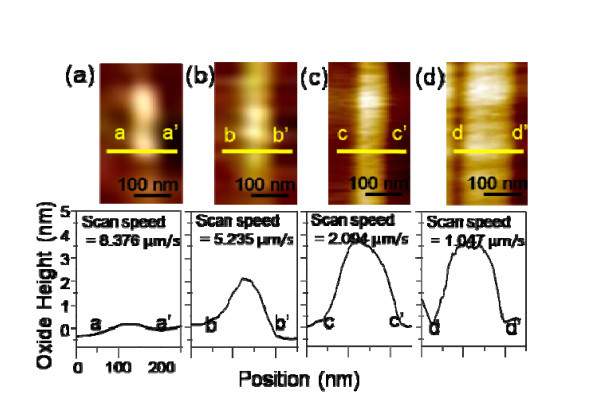
**AFM images and cross-sectional curves of oxide lines on m-plane 4H-SiC obtained under different scan speeds: (a) 8.376 μm/s; (b) 5.235 μm/s; (c) 2.094 μm/s; and (d) 1.047 μm/s**.

Then, the effect of the scan speed on the different crystalline plane orientations was investigated The AFM-LO was performed on a-, c-, and m-plane 4H-SiC wafers with an applied voltage of 10 V (tip as a cathode) under different scan speeds of 8.376, 5.235, 2.094, and 1.047 μm/s. Figure 4 presents typical AFM topography images of the four sets of oxide lines obtained by AFM-LO on a c-plane 4H-SiC wafer. The oxide height profile of Figure 3 shows that the local oxidation is enhanced by decreasing the scan speed. As shown in Figure 4a, d, a lower scan speed (1.047 μm/s) favors oxide line formation (17.17 nm), while a higher scan speed (8.376 μm/s) leads to depressed oxidation (3.34 nm). Figures 5 and 6 show the AFM topography images of the four sets of oxide lines obtained by AFM-LO on a- and m-plane 4H-SiC wafers, respectively. The AFM-LO as a function of scan speed on a- and m-plane 4H-SiC is similar to that of scan speed on c-plane 4H-SiC. The local oxidation on a-plane 4H-SiC is also improved by lowering the scan speed, although the tendency for this is minimized. In the case of a lower scan speed (1.047 μm/s), the oxide height increases (3.33 nm), while a higher scan speed (8.376 μm/s) leads to a lower oxide height (1.41 nm), as shown in Figure 5a, d, respectively. Figure 6a shows an oxide line pattern having an oxide height of 4.08 nm with a lower scan speed (1.047 μm/s). The higher scan speed (8.376 μm/s) leads to a lower oxide height (0.79 nm), as shown in Figure 6d. The AFM-LO is improved by the lower scan speed, which causes the duration of the applied voltage to be longer [17].

**Figure 7:**
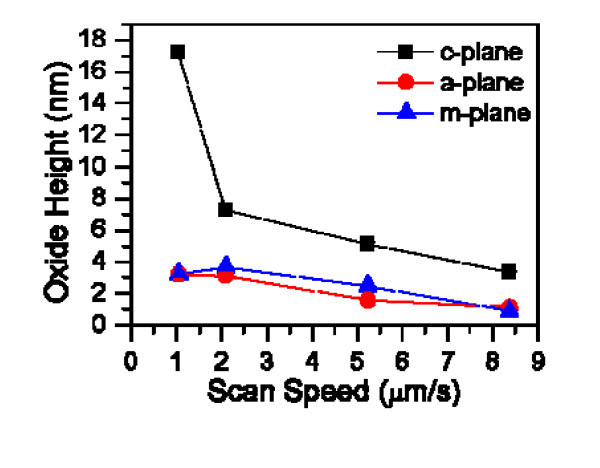
**Oxide height as a function of scan speed on different 4H-SiC by AFM-LO**.

These results are shown in Figure 7, where the oxide heights versus the scan speed on a-, c-, and m-planes of 4H-SiC are compared. The oxide height decreases as the scan speed increases on all a-, c-, and m-planes of 4H-SiC, suggesting that a longer anodization time resulted in an increased oxidation rate. It has clearly been shown that the AFM-LO rate on c-plane 4H-SiC is significantly higher than the other plane orientations, which may be related to the areal density of the first layer for the different surfaces.

Table 1 shows the oxdiation rates for both AFM-LO and thermal oxdiation on the three different plane orientation of 4H-SiC as well as the doping concentration and the theoretical planar atomic density values. The c-plane surface has much more carbon areal density than a- and m-plane surfaces and the theoretical planar atomic density of the c-plane (12.17) is higher than that of the a-plane (7.45) and m-plane (6.42) of 4H-SiC, as shown in Table 1. It can be seen that the oxidation rate is mainly proportional to the carbon areal density [18], and the enhanced thermal and local oxidation rates on c-plane 4H-SiC is ascribed to the high planar atomic density. However, the oxide height of the a-plane (6.5 nm) seems to be lower than that of the m-plane (13 nm), even though the planar atomic density of the a-plane (7.45 atoms/cm^2^) is higher than that of the m-plane (6.42 atoms/cm^2^). This may be related to the different doping concentration values for a- (*N*_D_: 5.9 × 10^18 ^cm^-2^) and m- (*N*_D_: 9.3 × 10^18 ^cm^-2^) plane-oriented samples,, because the effective electric field value is increased at higher doping levels, as shown in the simulation results in Figure 2.

**Table 1:** Oxidation rates of AFM-LO and thermal oxidation, as well as theoretical planar atomic density at three different plane orientations of 4H-SiC orientations with doping concentration profiles of 4H-SiC

Process	Oxide height (nm)		
	
	a-plane 4H-SiC	c-plane 4H-SiC	m-plane 4H-SiC
Thermal oxidation (nm)	109.1	153.7	81.1
Planar atomic density (atoms/cm^2^)	7.45	12.17	6.42
Local oxidation (nm)	6.5	30	13
Doping concentration (×10^18 ^cm^-2^)	5.9	9.6	9.3

## Conclusions

In conclusion, the effects of crystalline plane orientations of a-, m-, and c-planes on the AFM-LO of 4H-SiC wafers were investigated. It has been shown that the AFM-LO oxide heights of a-plane and m-plane 4H-SiC are lower than that of c-plane due mainly to the difference of planar density. It has clearly been shown that the AFM-LO rate on c-plane 4H-SiC is significantly higher than the other plane orientations, which can be correlated to the areal density of the first layer for the different surfaces as well as the doping concentration. The oxide height decreases as the scan speed increases, which suggests that a longer anodization time resulted in increased oxidation rates.

## Abbreviations

AFM: atomic force microscopy; AFM-LO: AFM-based local oxidation; SiC: silicon carbide.

## Competing interests

The authors declare that they have no competing interests.

## Authors' contributions

JJA and YDJ carried out the local oxidation experiments. SCK and JHL participated in analyzing the experimental data and calculations. JJA prepared the manuscript initially. SMK conceived of the study, and participated in its design and coordination. All authors read and approved the final manuscript.
